# Workshop report. Circadian rhythm sleep–wake disorders: gaps and opportunities

**DOI:** 10.1093/sleep/zsaa281

**Published:** 2021-02-14

**Authors:** Jeanne F Duffy, Sabra M Abbott, Helen J Burgess, Stephanie J Crowley, Jonathan S Emens, Lawrence J Epstein, Karen L Gamble, Brant P Hasler, David A Kristo, Roneil G Malkani, Shadab A Rahman, S Justin Thomas, James K Wyatt, Phyllis C Zee, Elizabeth B Klerman

**Affiliations:** 1 Division of Sleep and Circadian Disorders, Department of Medicine, Brigham and Women’s Hospital and Harvard Medical School, Boston, MA; 2 Department of Neurology, Northwestern University Feinberg School of Medicine, Chicago, IL; 3 Department of Psychiatry, University of Michigan, Ann Arbor, MI; 4 Department of Psychiatry and Behavioral Sciences, Rush University Medical Center, Chicago, IL; 5 Department of Psychiatry, Oregon Health & Science University, Portland, OR; 6 Department of Psychiatry University of Alabama at Birmingham, Birmingham, AL; 7 Department of Psychiatry, University of Pittsburgh School of Medicine, Pittsburgh, PA; 8 Department of Neurology, Massachusetts General Hospital and Harvard Medical School, Boston, MA

**Keywords:** actigraphy, advanced sleep–wake phase disorder, bright light therapy, chronotype, circadian amplitude, circadian period, circadian phase, circadian rhythm sleep–wake disorders, delayed sleep–wake phase disorder, dim light melatonin onset, entrainment, melatonin, non-24-hour sleep–wake disorder, irregular sleep–wake rhythm disorder

## Abstract

This White Paper presents the results from a workshop cosponsored by the Sleep Research Society (SRS) and the Society for Research on Biological Rhythms (SRBR) whose goals were to bring together sleep clinicians and sleep and circadian rhythm researchers to identify existing gaps in diagnosis and treatment and areas of high-priority research in circadian rhythm sleep–wake disorders (CRSWD). CRSWD are a distinct class of sleep disorders caused by alterations of the circadian time-keeping system, its entrainment mechanisms, or a misalignment of the endogenous circadian rhythm and the external environment. In these disorders, the timing of the primary sleep episode is either earlier or later than desired, irregular from day-to-day, and/or sleep occurs at the wrong circadian time. While there are incomplete and insufficient prevalence data, CRSWD likely affect at least 800,000 and perhaps as many as 3 million individuals in the United States, and if Shift Work Disorder and Jet Lag are included, then many millions more are impacted. The SRS Advocacy Taskforce has identified CRSWD as a class of sleep disorders for which additional high-quality research could have a significant impact to improve patient care. Participants were selected for their expertise and were assigned to one of three working groups: Phase Disorders, Entrainment Disorders, and Other. Each working group presented a summary of the current state of the science for their specific CRSWD area, followed by discussion from all participants. The outcome of those presentations and discussions are presented here.

**Statement of Significance**

This article results from a workshop in which sleep and circadian researchers and clinicians met to identify existing gaps in diagnosis and treatment, and areas of high-priority research for circadian rhythm sleep–wake disorders (CRSWD). CRSWD are a distinct class of sleep disorders in which the timing of sleep is either earlier or later than desired, irregular from day-to-day, and/or occurs at the wrong circadian time. While there has been enormous progress in understanding the molecular and cellular mechanisms of circadian rhythmicity and knowledge about normal human circadian physiology, little of this knowledge has been translated into clinical practice and our understanding of CRSWD remains severely lacking. This article summarizes the current state of the science and knowledge gaps for CRSWD.

## Executive summary

This White Paper presents the results from a workshop whose goals were to bring together sleep clinicians and sleep and circadian rhythm researchers to identify: (1) existing gaps in diagnosis and treatment and (2) areas of high-priority research in circadian rhythm sleep–wake disorders (CRSWD). The “Circadian Rhythm Disorders Workshop” was sponsored by the Sleep Research Society (SRS) and the Society for Research on Biological Rhythms (SRBR) and was held on June 8, 2019, prior to the SLEEP 2019 meeting in San Antonio, TX.

CRSWD are a distinct class of sleep disorders “caused by alterations of the circadian time-keeping system, its entrainment mechanisms, or a misalignment of the endogenous circadian rhythm and the external environment”. In these disorders, the timing of the primary sleep episode is either earlier or later than desired, irregular from day-to-day, and/or sleep occurs at the wrong circadian time. Pathophysiology can occur at the level of input to the suprachiasmatic nucleus (SCN, the central circadian pacemaker) or within the SCN itself, resulting in reduced amplitude or mistiming of rhythms. Symptoms can be further worsened when the individual is in an environment with limited or irregular time cues, as seen with institutionalized patients. In addition to abnormalities of the circadian system, other etiological factors may include alterations of sleep homeostatic mechanisms and behavioral choices, which in turn interact with the circadian system. While there are incomplete and insufficient prevalence data, CRSWD likely affect at least 800,000 and perhaps as many as 3 million Americans, and if Shift Work Disorder and Jet Lag are included then many millions more are impacted. The SRS Advocacy Taskforce has identified CRSWD as a class of sleep disorders for which additional high-quality research could have a significant impact to improve patient care.

Given those two goals, the specific topics that the workshop attendees were charged to address were:

1) The current state of the science for each disorder, including what is known about the causes/mechanisms underlying the disorder, the natural history of the disorder, and the prevalence of the disorder.
2) The adequacy of existing diagnosis and treatment guidelines for the disorder, including the specificity of phenotyping/diagnostic procedures, and the availability of robust biomarkers or other tools for diagnosis.
3) Knowledge gaps related to the pathophysiology, diagnosis, and/or treatment for the disorder. This may include additional research that is needed; new diagnostic tools/biomarkers that need to be developed; whether new treatments are needed; and whether patient registries, common data collection tools, and/or multisite collaborations are warranted. Furthermore, what gaps could be addressed with our current knowledge and tools, and what new tools need to be developed for other gaps to be addressed.
4) The “greater picture” for expanding the impact from the sleep-circadian community to general medicine/public health/education/productivity and other areas.

Participants were selected to represent a mixture of clinicians (3), clinician-researchers (11), and clinical researchers (6) or from the National Institutes of Health (NIH; 2) and were members of the SRS, SRBR, and/or American Academy of Sleep Medicine (AASM). The participants were selected for their expertise in one of three CRSWD areas and were assigned to one of three working groups: Phase Disorders, Entrainment Disorders, and Other. At the meeting, each working group presented a summary of the current state of the science for their specific CRSWD area, followed by discussion from all participants. There was then time for working sessions within each group, followed by a second presentation from each group and group discussion.

Each of the working groups carried out a review of the literature prior to the workshop and again while drafting this White Paper. Scientific databases (i.e. PubMed and Google Scholar) were searched for the key words describing and related to CRSWDs and the resulting papers were reviewed for relevant content. In the case of the “circadian rhythm sleep disorder not otherwise specified” group, that term did not yield any articles. Therefore, the circadian rhythm disorder not otherwise specified working group had their participants provide research articles (both basic science and clinical research on other medical and psychiatric disorders) that might be relevant to this diagnostic category to aid in the discussions and drafting of this White Paper. Because Shift Work Disorder is already the focus of extensive research, the organizers chose to focus on other CRSWDs.

In this summary of the workshop, we begin with common themes across all CRSWD, followed by specifics of each CRSWD type.

## Introduction

**Figure 1.**
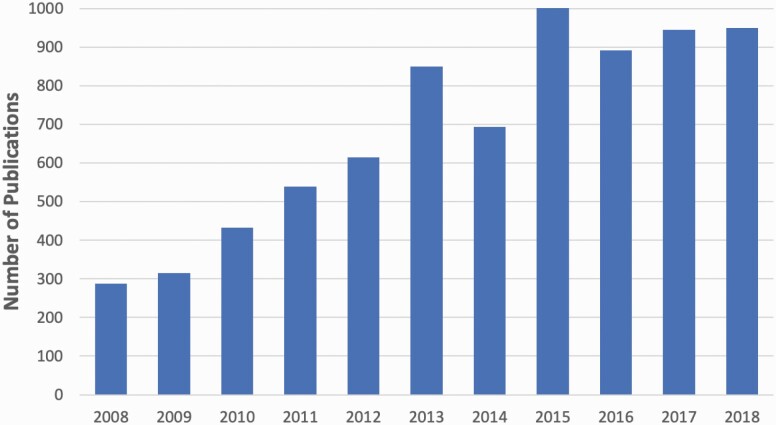
Publications resulting from NIH grant support with “circadian” as key word. Each bar represents the number of publications in that year that had the key word “circadian” and were supported by NIH grants. The number of such NIH-supported publications tripled across the first half of the decade and then remained stable thereafter. In the decade from 2008 to 2018, there were a total of 7,525 publications with the key word “circadian” that were supported by NIH grants. While this explosion of knowledge has revealed much about the workings of the circadian system and some of the consequences of disrupted circadian rhythmicity, relatively few grants and resulting manuscripts have focused on the pathophysiology or prevalence of CRSWDs or on testing new treatments for them.

Our overall knowledge about the circadian timing system has been rapidly expanding since the suprachiasmatic nucleus (SCN) was identified as the central circadian pacemaker in mammals in the early 1970s [1–3]. We have learned that there is a direct pathway from intrinsically photosensitive retinal ganglion cells (ipRGCs) in the eye to the SCN to transmit light–dark information from the environment to the clock [4], that individual SCN neurons show circadian rhythms in their electrical firing rate [5], and that the 24-h rhythms are also present in most cells of the body due to a transcriptional–translational “molecular clock.” Importantly, the human circadian system shows properties similar to that of other mammals in its organization. As presented at the Workshop, in just the past decade, there have been more than 7,500 publications with the key word “circadian” that have been supported by more than 450 NIH grants (Figure 1).

Despite this explosion of knowledge about normal circadian physiology, the consensus from the Workshop is that our understanding of CRSWD is currently severely lacking in multiple areas and little of what we have learned about basic circadian physiology has been translated into clinical practice. Below, we summarize the current state of the science and knowledge gaps for CRSWD.

## Overall gaps across all CRSWD

CRSWD are characterized by sleep and wakefulness occurring at abnormal or irregular times. In shift work and jet lag, this is due to specific behaviors that result in sleep occurring at an adverse circadian time. Attempting to sleep during the biological daytime and to remain awake during the biological nighttime typically results in disrupted and shortened sleep and excessive sleepiness during the planned wake episode. The working group made a decision to not address jet lag disorder or shift work disorder in the workshop or in this report, although we do believe they warrant a similarly focused critical examination.

In the remaining CRSWDs, it is assumed that the abnormal or irregular timing of sleep is due to an abnormality in the underlying circadian system. However, as is discussed in detail later, because assessment of circadian rhythms is not currently required to diagnose or treat CRSWD, in most cases, the underlying circadian defect is unknown. Furthermore, studies in which the circadian rhythms of patients with CRSWD have been measured have provided evidence that in some cases, the timing of the patient’s circadian rhythms appear normal, despite the abnormal sleep timing.

The major gaps for all these disorders are:

1) General lack of information about cause, prevalence, time course, and comorbidities. CRSWD symptoms or diagnoses have neither been included in large population-level cohorts that include diverse samples of individuals, nor in longitudinal cohorts that link circadian disorders with health outcomes.
2) Assumption that a defect in the circadian system is an underlying cause in CRSWD without assessment of circadian timing or aspects of circadian physiology for diagnosis or treatment. While this is largely due to a lack of understanding of the pathophysiological basis of CRSWD combined with a lack of practical diagnostic procedures (see later), it likely results in inappropriate treatments in some patients [6] and may be responsible for the lack of efficacy in many patient groups. In addition, there is some evidence that in at least some patients, the timing of their circadian phase appears normal despite abnormal sleep timing [6].
3) Reliance on sleep timing (e.g. sleep diaries or actigraphy) for diagnosis or treatment. As noted earlier, there are documented cases where underlying circadian timing is normal despite extreme sleep timing [6]. There is also accumulating evidence that other behaviors (e.g. self-selected light exposure), sensitivity to circadian-relevant light stimuli, and even sleep–wake homeostatic dynamics may play a role in some patients and may need to be evaluated for complete diagnosis and treatment.
4) Lack of practical (e.g. short, easy to carry out, inexpensive) markers of central and peripheral circadian rhythm timing. While actigraphy is easily available and provides an objective assessment of the timing of rest, sleep, and activity, there are no widely available methods for accurately determining circadian timing in patients with suspected CRSWD. While some clinics have implemented collection of salivary dim light melatonin onset (DLMO, a measure of circadian phase) in the clinic or in patients’ homes [7, 8], this method is time-consuming, expensive (and not covered by medical insurance), and lacks established guidelines for normal values. The emergence of transcriptome, metabolome, proteome, or biomarker panels that require only one or two blood samples or the use of actigraphy plus another longitudinal signal may eventually provide alternatives to DLMO [9–12]. However, validation of these biomarkers in studies where sleep timing is altered, in field studies, and in patient populations will be an important precursor to clinical implementation.
5) Lack of proven efficacious treatments, guidelines for treatment, and skilled health care providers for diagnosing and treating patients. Current treatments may not necessarily be tailored to underlying physiology when that physiology remains unknown. Once physiology is known, novel treatments that target as-yet unknown abnormalities (e.g. within the retina or SCN) may be tested. For example, if a phase disorder is determined to be largely the result of altered light sensitivity [13] driven by altered function of ipRGCs (which mediate nonvisual responses to light) [14], or altered downstream processing of ipRGC responses, then novel treatments directly targeting ipRGC response to mitigate the altered physiology may be useful as an adjunct to, or in place of, conventional light therapy and/or melatonin supplementation. Current treatments have a high failure rate, and it is often unclear whether that is due to lack of efficacy or to lack of adherence on the part of the patient. Because the barriers to adherence are not known, we cannot yet develop strategies to overcome these barriers. For example, the efficacy of bright light therapy for DSWPD is contingent upon the patient waking up on time to use the device—in essence, a “Catch-22” wherein the patient must overcome one of the primary challenges of the disorder (waking up on time) in order to implement the treatment aimed at addressing that symptom. Furthermore, while such behavioral and motivational barriers to adherence are presumed, they have not been systematically assessed and characterized. Relatedly, methods for effectively tracking adherence to light therapy remain limited (although see [15] for one promising approach). Furthermore, the patient’s subjective experience should be considered when evaluating treatment success, and this has been lacking in prior research [16]. For example, sleep/circadian improvements such as correcting phase alignment, improving sleep continuity, and extending sleep duration do not always clearly correspond to reductions in daytime fatigue or improvements in mood. Finally, while it is assumed that interventions using light and melatonin are relatively safe, long-term safety monitoring of retinal health in all patients using light emitting devices, particularly monochromatic short wavelength (blue) light, and endocrine markers and risk of type 2 diabetes mellitus in children/adolescents and genetically susceptible individuals who are treated with melatonin, as well as minimum effective dosing for light and melatonin, are still needed.

## Phase disorders

Section participants: Brant P. Hasler (leader), Stephanie J. Crowley, Charles A. Czeisler, Karen L. Gamble, Shadab A. Rahman, and S. Justin Thomas.

**Figure 2.**
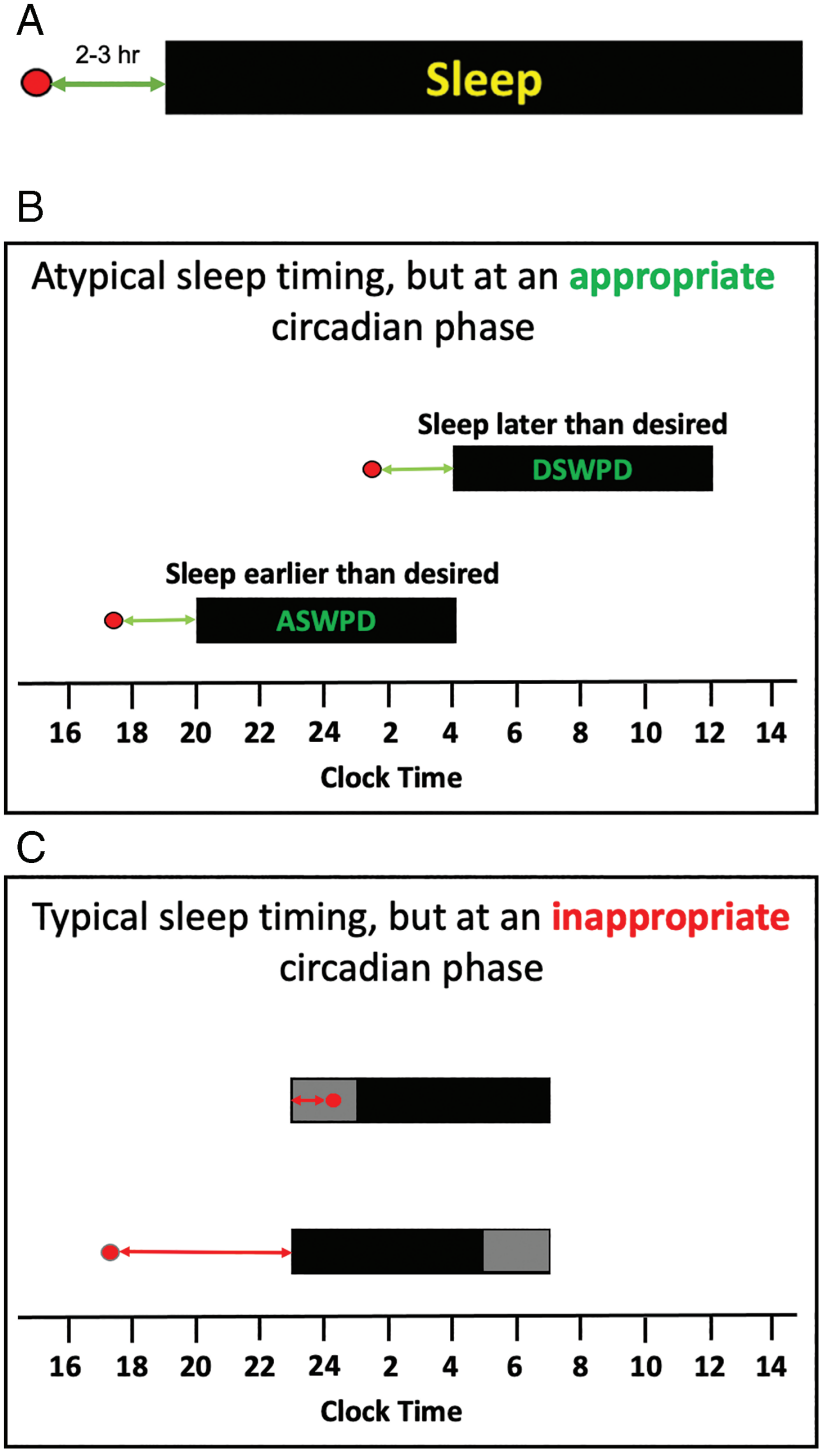
Relationship between the timing of the circadian phase of the DLMO and the timing of the nocturnal sleep episode. For all panels, the *x*-axis represents time; DLMO phase is indicated by the red circle; the grey bar represents time in bed, and the black bar represents sleep. (A) Typical phase relationship (in postpubertal adolescents and adults). (B) Circadian rhythm sleep–wake phase disorders are assumed to be caused by an “early” (in the case of ASWPD) or “late” (in DSWPD) “circadian phase timing” which in turn results in sleep occurring earlier or later than desired. In the cases illustrated here, the relative timing between the circadian phase of DLMO (red circle) and sleep onset is appropriate (i.e. sleep occurs at an appropriate circadian time), but the clock time at which each occurs is inappropriate (see also Figure 3, A). (C) Circadian rhythm sleep–wake phase disorders can also arise when the “relative timing” between the underlying rhythm of sleep–wake propensity is “inappropriately aligned relative to” the timing of sleep. This can occur even if time in bed is at a conventional/desired time. This may result in a prolonged sleep latency (indicated by the gray shading at the beginning of scheduled sleep in the upper sleep bar) or early morning awakening (indicated by the gray shading at the end of the sleep episode in the lower bar; see also Figure 3, B and C).

The human circadian timing system produces a daily rhythm of sleep–wake propensity, and appropriate timing between this intrinsic rhythm and the timing of behavior is critical to allow for consolidated sleep and wake [17]. Under normal conditions, sleep is initiated in the late evening, typically 2–3 h after the onset of melatonin secretion (see Figure 2, A). CRSWD of phase are broadly defined as inappropriately timed sleep due to inappropriate timing of this intrinsic circadian rhythm of sleep–wake propensity [18]. Thus, in advanced sleep–wake phase disorder (ASWPD), the underlying assumption is that the phase of the circadian system is earlier than the desired sleep–wake times, resulting in difficulty staying awake until the desired bedtime and a corresponding early morning awakening [18]. Similarly, in delayed sleep–wake phase disorder (DSWPD), the assumption is that the phase of the circadian system is later than the desired sleep–wake times, resulting in difficulties falling asleep and waking up when desired [18] (see Figure 2, B). However, current diagnosis of ASWPD or DSWPD requires only that the timing of “sleep” is earlier or later than desired, recommending but not requiring that underlying “circadian timing” is even assessed.

**Figure 3.**
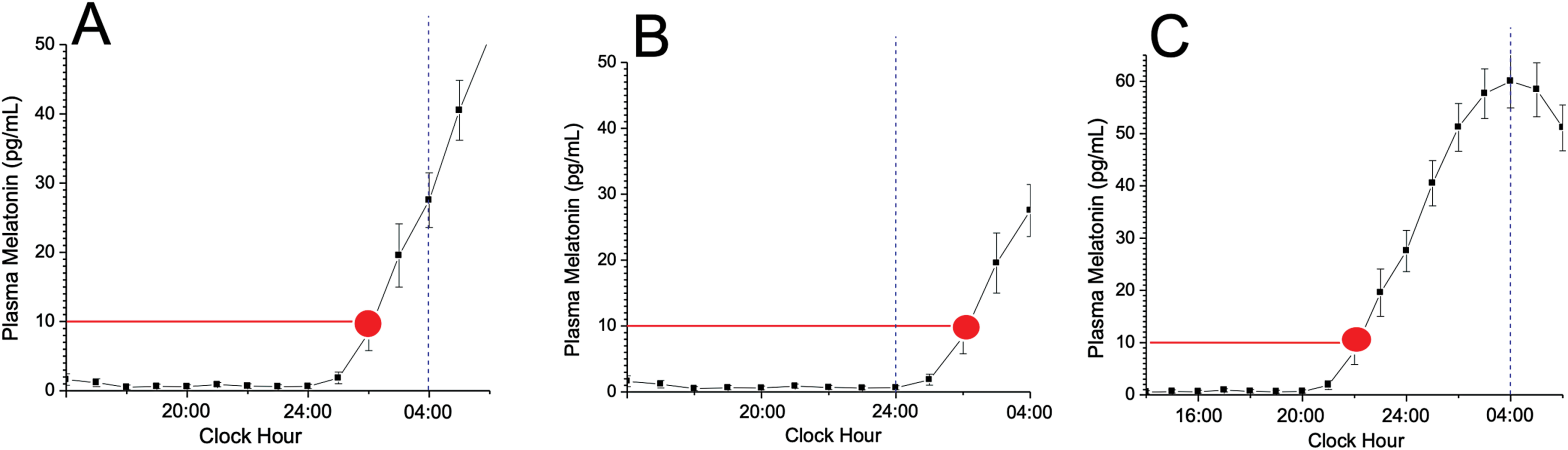
Illustration of the complexity of CRSWD. Melatonin data are plotted with respect to clock time; red circle indicates time of DLMO; dashed blue vertical line represents bedtime. (A) “Classic” DSWPD in which the timing of the circadian system and the timing of sleep are both occurring later than normal (circadian phase of DLMO ~02:00 am, bedtime ~04:00 am; see also Figure 2, B). (B) A case where bedtime is at a conventional hour (midnight) but DLMO is occurring later than normal (~02:00 am). Such a patient would likely experience prolonged sleep latency and may be misclassified as having sleep onset insomnia, rather than DSWPD. (C) A case where DLMO is occurring at a conventional hour (~10:00 pm) but bedtime is occurring much later than normal (~04:00 am). According to current standards, such a patient would not have had their DLMO assessed in the process of being diagnosed as having DSWPD. In this case, treatments designed to shift circadian phase earlier (morning light, evening melatonin) may be ineffective, given that circadian phase is already occurring at a conventional time, despite the fact that sleep is not occurring at an appropriate circadian phase (see also Figure 2, C).

Although the pathophysiology in both ASWPD and DSWPD is hypothesized to be due to a misaligned circadian phase [19], recent studies that have measured phase in patients with ASWPD and DSWPD have found that an altered circadian phase may not always be present [6, 8, 19], and the sleep timing may be advanced or delayed without a corresponding shift in the underlying circadian timing. For example, two groups have independently found that DSWPD patients can be broadly dichotomized into two distinct subtypes based on their endogenous circadian phase [6, 15]: those patients with both delayed sleep and delayed endogenous circadian phase (Figure 3, A), and those with delayed sleep but without a delayed endogenous circadian phase (Figure 3, C). These recent findings highlight that there must be multiple underlying mechanisms driving the delayed sleep phenotype.

In contrast to these circadian rhythm disorders that arise from a mismatch between intrinsic and extrinsic factors, both jet lag disorder and shift work disorder are due to extrinsic factors (i.e. travel and work schedules, respectively) that result in misalignment between the sleep–wake times and the intrinsic circadian rhythm [18]. As noted above, in this article, we will not discuss them and will focus on ASWPD and DSWPD.

### Current state of the science

Few studies have examined the incidence and prevalence of ASWPD or DSWPD. While self-reports of ASWPD range up to 7% [20], prevalence estimates using International Classification of Sleep Disorders (ICSD) criteria range from 0% [21] to 0.21% [22]. The true prevalence of ASWPD may be underreported, in part because the tendency towards early sleep timing is less problematic for conventional academic/work schedules, and because ASWPD may be most common among older adults who are often retired and therefore may have more flexible schedules. Similarly, the prevalence of DSWPD is “poorly documented in the empirical literature” [23], with estimates ranging from 0.2% to 16% depending on varied operationalizations of the criteria and age group studied [21, 24–26]. Consistent with lifespan trends for chronotype [27], DSWPD onset commonly occurs during adolescence and young adulthood, and may remit during later adulthood given the reported lower prevalence among middle-aged adults [28]. Surveys conducted by one patient advocacy group, however, suggest that DSWPD may, in some cases, worsen with age [16].

### Etiology and pathophysiology

The etiologies underlying ASWPD and DSWPD remain poorly understood. Genetic contributions have been documented for familial forms of ASWPD, implicating autosomal-dominant inheritance involving PER2 and CSNK1D [29–31]. Evidence for genetic contributions to DSWPD remain confined to a single report of a CRY1 mutation [31], although it is plausible that apparent genetic links to eveningness more broadly (e.g. PER genes) [32, 33] may also be relevant to both ASWPD and DSWPD [34].

Proposed etiological explanations for DSWPD with empirical evidence include circadian phase delay [6, 19, 35], long circadian period [36], reduced light sensitivity in the morning or increased light sensitivity in the evening [14, 37], specific daily light exposure patterns [38, 39], and alterations in sleep–wake homeostatic dynamics [40]. Notably, these etiologies are not mutually exclusive.

### Existing diagnosis and treatment guidelines

Diagnoses of ASWPD and DSWPD are currently based on a clinical interview in combination with sleep logs, and they require at least 3 months of difficulty falling asleep and awakening at the desired time along with an advance or delay of the habitual sleep episode as demonstrated by a minimum of 7 days of sleep logs [14]. At least 14 days of actigraphy are also recommended but not required, as are self-report questionnaires assessing chronotype or morningness–eveningness, or even better, physiological markers of circadian phase (e.g. DLMO) when available (though rarely collected in practice). We recommend the standardization of a minimum of 14 days of sleep logs and/or actigraphy across all of the CRSWDs, an increase from the 7-day minimum recommended in the diagnostic criteria for the Phase Disorders. A minimum of 14 days of data collection has the advantage of capturing sleep–wake patterns over 2 weekends or non-work days and, thus, would provide a more complete view of the patient’s sleep–wake activity when unconstrained by work or school. The primary differential diagnoses of both ASWPD and DSWPD include other sleep disorders (i.e. insomnia) and psychiatric disorders.

### Phenotyping and diagnosis

Current phenotyping and diagnostic procedures for ASWPD and DSWPD are insufficient for several reasons. First, the use of relative (i.e. to desired schedule) versus fixed (i.e. fixed clock time) temporal cutoffs for defining advanced or delayed timing is problematic. For example, theoretically, an individual exhibiting a sleep–wake schedule of 11:00 pm–07:00 am could be diagnosed with ASWPD or DSWPD if that schedule were considered too early or late relative to their “desired” schedule. Although we recognize that this individual would not likely be diagnosed with either ASWPD or DSWPD, this extreme example underscores a potential problem with the relative (i.e. desired schedule) temporal cutoff in the current definitions. The heterogeneity of operationalized diagnostic criteria in research studies (e.g. [26, 41]) has likely contributed to the variability in prevalence estimates.

Second, as stated above, the lack of objective circadian measurements may lead to the improper application of treatments that are designed to alter circadian phase (e.g. bright light therapy and chronotherapeutic use of melatonin) when other treatments (e.g. cognitive behavioral therapy for insomnia [CBT-I] and/or hypnotic medications) might be more appropriate. The current evidence base underlying actigraphy’s use in ASWPD or DSWPD diagnosis remains scant. A systematic American Academy of Sleep Medicine (AASM) review asserted that actigraphy is a “very useful tool for assessing circadian dysrhythmia”; this assertion is based on a total of five studies (only three of which related to delayed phase) described as having “very low to low” evidence [42].

Third, the current diagnostic procedures may not sufficiently distinguish DSWPD from insomnia disorder and/or frequently comorbid psychiatric disorders, in particular, depressive disorders. An estimated 10% of patients diagnosed with chronic insomnia disorder may be more appropriately diagnosed with DSWPD [43]. Significant depressive symptomatology appears to be present in half or more of individuals with DSWPD [19, 44, 45], and delayed phase is increasingly documented in association with substance use and other psychiatric disorders including ADHD [46] and schizophrenia [47], raising further questions about endogenous versus behaviorally driven delays in sleep and/or circadian timing.

For individuals with so-called “non-circadian” DSWPD (Figure 3, C), the physiology may not be that circadian phase per se is unusually late (and thus the phase relationship between circadian phase and sleep timing abnormally short) but that the phase relationship between circadian phase and sleep timing is abnormally “long”. In these cases, the light–dark cycle associated with late sleep/wake times would be expected to shift the circadian clock later so that a “normal” phase relationship is present. Why this is not occurring in some people with DSWPD (or ASWPD) who have “normal” circadian phases needs to be evaluated; homeostatic mechanisms (e.g. unusually long build-up of homeostatic pressure in DSWPD) should be considered and evaluated.

### Biomarker availability

As noted earlier, there is a lack of practical, robust biomarkers, or other tools for diagnosis of ASWPD and DSWPD. As critically reviewed in Dijk and Duffy [48], the emerging biomarker contenders remain limited by several factors, including questionable practicality in the clinic (e.g. two blood samples 12 h apart), insufficient comparison to the current gold standard circadian phase measure in humans (DLMO), and/or insufficient testing in populations who experience circadian misalignment—perhaps the most concerning limitation for the present discussion. There are also questions about reimbursement for these methods in the clinic; currently, the circadian clinics employing DLMO require patients to self-pay. With respect to behavioral assays, Dijk and Duffy [48] also review a promising approach applying mathematical models originally developed by Kronauer et al. [49] to predict DLMO based on activity and light data obtained from actigraphy. This approach appears to be able to have a sufficiently low margin of error (<2 h) to be useful for diagnosis and treatment planning, and thus far has been validated in a small sample of healthy men ranging in chronotype but sleeping on their habitual schedules [50], as well as among small groups of actual [51] and simulated [52] shift workers.

### Treatment guidelines

Current treatment guidelines for ASWPD and DSWPD remain inadequate. Bright light therapy, strategically timed melatonin, and chronotherapy (i.e. treatments designed to alter the timing of circadian rhythms) are the most widely recommended treatment approaches, but their evidence base remains insufficient. A 2015 update [53] of AASM clinical practice guidelines for treatment of “intrinsic” CRSDs, including ASWPD and DSWPD, described a dearth of high-quality evidence for these treatment approaches, reporting evidence that was “weak for” the efficacy of evening light therapy for adults with ASWPD, morning light therapy in conjunction with behavioral treatments for children/adolescents with DSWPD, and strategically timed melatonin for adults and children/adolescents with DSWPD with and without psychiatric comorbidities. Given the heterogeneity of treatment protocols and lack of published supporting evidence, no specific recommendations were made regarding the use of melatonin, light therapy, chronotherapy, or other treatment options (e.g. use of blue-blocking glasses). Based on our review of the DSWPD literature since the 2015 article [41, 54–57], the high degree of heterogeneity of treatment protocols (increasingly multicomponent), the inconsistent inclusion of DLMO or other biological circadian phase markers, and the inadequate use of appropriate control conditions, are a barrier to developing evidence-based treatment guidelines.

Insufficient availability of efficacious treatment further compounds the lack of definitive treatment guidelines for ASWPD and DSWPD. There are relatively few providers and clinics with expertise. Although light boxes are widely available with falling prices, and wearable light devices exist with limited, if promising evidence bases [58–60], patients and providers do not have guidelines for their use. Furthermore, while exogenous melatonin is also widely available over the counter, insufficient consideration of administration timing, inappropriate dosages and formulations (e.g. extended release), unknown reliability of dose potency and purity [61], and limited safety data in children and adolescents [62] despite its common use [63], all raise serious concerns. Although prescription melatonin receptor agonists are presumably of higher quality and more reliable content, none are currently aimed at or approved for treating ASWPD or DSWPD. Furthermore, use of melatonin receptor agonists may inadvertently impact other physiological systems given the ubiquitous expression of melatonin receptors throughout the body [64]. A better understanding of possible contraindications is needed.

### Knowledge gaps specific to phase disorders

• Etiology and pathophysiology of ASWPD and DSWPD are poorly understood, but the few studies that have been carried out point to shorter circadian period related to family mutations in ASWPD, and longer circadian period, delayed phase, altered sleep–wake homeostatic responses, and changes in light sensitivity [13] as potential underlying mechanisms for DSWPD.
• Diagnostic criteria do not currently include direct assessment of circadian phase using biomarkers (e.g. DLMO). Subtypes of ASWPD and DSWPD with different etiologies appear likely [6, 8, 19], but have not been clearly characterized, preventing the development of appropriate treatments.
• The role of circadian amplitude is not understood and procedures to assess it are currently lacking.
• As described earlier, a common operationalized diagnostic process and criteria across clinics/research groups is lacking. Patient registries or multisite studies could accelerate the research needed to develop better diagnostic criteria.
• Behavioral assays that are practical and cost-effective need to be developed to better characterize ASWPD and DSWPD concurrent with biomarkers.
• Efficacy studies of existing treatments (e.g. light therapy, chronotherapy, and melatonin) are limited by small sample sizes at single sites, and nondiverse populations.

### Recommendations and future directions specific to phase disorders

To address these gaps in our knowledge and limitations to current treatments, future studies should include basic science approaches to studies in humans, translating what is currently known in animal models. In addition, clinical trials testing different treatment approaches, inclusion of diverse populations, and community and population-based cohort studies should be carried out. For example, basic studies are needed to investigate the different putative etiological factors, assessing individuals with these disorders along a spectrum of severity in terms of circadian period length, phase angle, homeostatic buildup during wake and dissipation during sleep, and both morning and evening light sensitivity. Ideally, such studies would include a range of rigorous assessment methods (and collected repeatedly over time) in order to simultaneously investigate, contrast, and compare the different putative etiological factors. These studies might then lead to the identification of subtypes with differing etiological bases. For patients with comorbidities, studies to determine the developmental time courses of the phase disorder and the other comorbid conditions are also needed. In order to develop a standard, operationalized diagnostic process, criteria should be expanded to include circadian phase and amplitude assessment, with a focus on identifying practical, cost-effective diagnostic tools such as behavioral and biomarker assays. In order to allow better generalizability of treatment methods, large-scale, multisite randomized clinical trials are needed in diverse populations. In the clinic, treatments should be expanded to include other targets beyond circadian phase (e.g. motivational enhancement to improve adherence, as well as targeting the homeostatic system). Lastly, the efficacy of existing treatments (e.g. light therapy, chronotherapy, and exogenous melatonin administration) need to be confirmed with studies that are adequately powered for generalization, followed by field studies to ascertain effectiveness. Although multicomponent treatments have become increasingly popular, including existing treatments targeting the circadian system into other modalities (e.g. CBT-I) may be premature and would then require dismantling studies to determine the effectiveness of each individual component and its impact on circadian phase.

## Entrainment disorders

Section participants: Sabra M. Abbott (leader), Helen J. Burgess, Lawrence J. Epstein, David A. Kristo, Roneil G. Malkani, and James K. Wyatt.

In contrast to phase disorders where there is a relatively stable timing of sleep from one night to the next and the assumption of stable timing of circadian rhythms, entrainment disorders are characterized by an instability of sleep timing from night-to-night. There are two major entrainment-related circadian sleep disorders: non-24-hour sleep–wake disorder (N24SWD) and irregular sleep–wake rhythm disorder (ISWRD). Because they have few similarities, they are presented separately within this section.

### Non-24-hour sleep–wake rhythm disorder

#### Current state of the science

N24SWD is characterized by an inability to entrain to a 24-h schedule. Typically, patients exhibit a progressive phase delay in circadian rhythms [65] although progressive phase advances may occur depending on the period length (tau) of the patient’s internal circadian pacemaker. In blind patients with N24SWD who try to maintain a stable sleep–wake schedule, daytime sleep and hypersomnolence can periodically occur along with nocturnal insomnia as a result of the internal rhythms drifting in and out of synchrony with the external 24-h day [53]. Almost half of blind patients without light perception may have N24SWD [66]; they develop N24SWD due to a lack of photic input via the retinohypothalamic tract to the circadian pacemaker in the SCN. The prevalence of N24SWD in sighted people is unknown. It is unclear why among blind individuals, some develop N24SWD, while others are able to maintain entrainment despite lack of light perception. Two reasons may be: (1) the ability of non-photic cues (e.g. exogenous agents [alcohol, caffeine, prescription or non-prescription medications], variably timed social and exercise stimuli, and variably timed rest-activity schedules) to entrain those blind individuals who have an endogenous circadian period close to 24 h [67] and (2) retained circadian photoreception in some blind individuals [68–70].

N24SWD can also develop in sighted individuals and may represent an extreme form of DSWPD, with DSPWD comprising a premorbid symptom for N24SWD in sighted individuals [71]. There is no known genetic marker or trigger for N24SWD (with the exception noted later [72]). Potential etiologies for N24SWD include a long intrinsic period, alterations in light sensitivity [13], and/or altered rates of buildup of homeostatic pressure, all potentially exacerbated by decreased structured social and physical activity [36]. In addition, there are case reports of N24SWD induced through chronotherapy [72], after the treatment strategy of purposefully delaying sleep–wake rhythms as a treatment for DSWPD [73].

Case reports of N24SWD following traumatic brain injury (TBI) exist, illustrating a need for vigilance to circadian sleep–wake dysfunction following TBI [74]. If left untreated, N24SWD is associated with the development of major depression, potentially as a result of the considerable disruption of normal daily activities [75].

Studies of both blind and sighted patients with N24SWD have the potential for improving our understanding of multiple areas of physiology. Sighted individuals with N24SWD provide a unique opportunity to better understand the underlying mechanisms of entrainment. Comparing results from sighted individuals with and without N24SWD may help determine which zeitgebers are essential for entrainment, and the period length that limits the ability to entrain to a 24-h day. In addition, if it is determined that sighted individuals with N24SWD do not respond appropriately to the common treatments of timed artificial bright light [13] or exogenous melatonin administration, research on other treatments could be pursued. Further study of entrainment strategies in blind individuals with N24SWD may lead to improved understanding of human circadian entrainment to non-photic zeitgebers in the general population. Many of these patients subjectively report symptoms consistent with a misalignment between peripheral oscillators (e.g. different sleep, feeding, and mental alertness rhythms). Confirming and studying the health consequences of the suspected internal circadian misalignment within this population may provide further insight into the general health consequences of circadian misalignment. These observations also suggest that strategies used to synchronize these peripheral oscillators may be beneficial.

### Existing diagnosis and treatment guidelines

The current diagnostic criteria for N24SWD focus on the use of daily sleep logs and wrist actigraphy for at least 14 days plus at least 3 months of self-reported clinical symptoms consisting of alternating periods of insomnia and hypersomnia followed by asymptomatic periods, depending on the current degree of alignment with the 24-h light/dark cycle [18]. Note that many individuals with N24SWD have times when they appear to be entrained, alternating with times when they exhibit a N24 pattern, so it can be challenging to adequately capture a N24 pattern with only 2 weeks of sleep logs or actigraphy data [73, 75]. Alternative proposed diagnostic tools include the use of serial biomarkers, such as multiple salivary DLMO or urinary 6-sulfatoxy melatonin (a metabolite of melatonin) measurements to confirm the N24 pattern and estimate the patient’s endogenous period [66]. Knowledge of circadian timing may also be useful to guide the timing of treatment. Current measures involve a significant amount of patient burden for their collection and are not usually paid for by medical insurance, resulting in a significant out-of-pocket expense for the patient.

Treatment recommendations for blind individuals with N24SWD, based on the results of several small studies, focus primarily on the use of timed melatonin (0.5–10 mg) or the melatonin agonist tasimelteon taken 1 h prior to desired bedtime [76–79]. For sighted individuals with N24SWD, the treatment options are far less clear, primarily due to a lack of large randomized controlled treatment trials within this population. Varying degrees of success have been achieved with combinations of light and melatonin for sighted patients with N24SWD [73, 80–82], although long-term compliance with these treatments can often be challenging [83]. As a result, the most recent AASM Clinical Practice Guidelines were not able to provide any evidence-based recommendations for the treatment of this sighted patients with N24SWD [53].

### Knowledge gaps specific to N24SWD

• Pathophysiology of N24SWD in blind individuals is not fully understood, since not all blind individuals are affected.
• Whether intrinsic circadian period (compared to observed period) plays a role in blind or sighted persons with N24SWD is not clear.
• Prevalence of N24SWD in sighted individuals is unknown.
• Whether altered sleep–wake homeostasis plays a role in N24SWD is unknown.
• Etiology, co-morbidities, and pathophysiology of N24SWD in sighted individuals are poorly understood.
• Whether and how TBI or other neurological or psychiatric disorders contribute to the underlying pathophysiology of this disorder remains unknown.
• Effective treatment strategies for N24SWD in sighted individuals are lacking.
• Effectiveness of non-photic zeitgebers (e.g. exercise, food intake, and social interactions) for entraining blind or sighted patients with N24SWD is unknown.
• The extent to which both external desynchrony (between the central clock and the environment) and internal desynchrony (between the central clock and peripheral clocks) exist in patients with N24SWD is unknown.
• How intermittent or long-term circadian misalignment impacts mental and physical health is poorly understood.

### Irregular sleep–wake rhythm disorder

#### Current state of the science

ISWRD is characterized by a lack of a 24-h sleep–wake pattern. People with ISWRD have insomnia, daytime sleepiness, or both due to difficulty sleeping during the desired sleep time, and multiple daytime naps, with the total amount of sleep being normal for the person’s age [18]. The lack of fixed rhythm results in a mostly unpredictable pattern of sleep and wake occurring throughout the 24-h day, though many patients will have a slightly longer sleep episode at night [84]. ISWRD is seen primarily in people with neurodevelopmental disorders [85–87], neurodegenerative disorders such as Alzheimer’s disease [88, 89], and psychiatric illnesses such as schizophrenia [90]. ISWRD has also been reported in people with TBI [91] and brain tumors affecting the hypothalamus [92].

The underlying pathophysiology of ISWRD is thought to be related to dysfunction of inputs to the SCN, the SCN itself, or its outputs. Some neurodegenerative disorders, such as Alzheimer’s disease, result in neuronal loss in the SCN, and the degree of SCN cell loss correlates with the reduction in rest-activity rhythm amplitude [93]. Current evidence also implicates impaired light signaling as a factor in the pathophysiology of ISWRD. People with developmental disorders involving optic nerve dysfunction [85] or congenital blindness [86] have reported ISWRD patterns, and people with Alzheimer’s disease can have pathology of the ipRGCs that provide light input to the SCN [94]. Output signals from the SCN can also be affected, resulting in impaired rhythmicity of some physiologic circadian rhythms and impaired feedback to the SCN. For example, there can be altered core body temperature regulation [95], decreased melatonin secretion [87, 96, 97], and decreased synchronization of brain rhythms [98].

Although the prevalence of ISWRD is unclear, it is more common in institutionalized older adults, especially those with Alzheimer’s disease [89]. The degree of dysfunction can progress over time, and the severity of ISWRD correlates with cognitive impairment [99] and dementia severity [88]. Alterations in circadian rhythms can also precede the development of cognitive impairment or dementia by several years [100].

### Existing diagnosis and treatment guidelines

The current diagnostic criteria for ISWRD require obtaining a complaint (or observation by a caregiver) of the inability to maintain consistent timing of sleep and wake episodes, with at least three irregularly spaced sleep episodes/ 24 h, for at least 3 months. Sleep diaries, ideally obtained in conjunction with wrist actigraphy, are required with a recording period of at least 7 days to confirm the diagnosis [18]. This disorder would not be diagnosed when these sleep–wake patterns are developmentally normal, such as in a young infant, or in patients with other sleep disorders (e.g. napping seen in hypersomnia).

Though the ICSD-3 requires that symptoms are not merely secondary to or better explained by another disorder, ISWRD is rarely seen in the absence of another disorder. As such it may be useful to consider this a “comorbidity” until more is known about this disorder.

Treatment options for patients with ISWRD consist of a variety of approaches. One intervention is to limit daytime intentional or unintentional sleep episodes [101] to sustain buildup of the sleep homeostatic drive until nighttime. Increasing daytime light exposure via phototherapy may improve problematic behavior in patients with dementia [102] but does not appear to improve nighttime sleep [103, 104].

The recent AASM Clinical Practice Guidelines recommend against the use of hypnotic medication (strong recommendation against) and melatonin (weak against) for treatment of ISWRD in older adults with dementia, though it did recommend (weak for) the use of melatonin in the treatment of ISWRD in children with neurological/neurodevelopmental disorders. It recommends phototherapy (weak for) and against melatonin plus phototherapy (weak against) treatment in older adults with dementia and ISWRD. It offers no recommendations regarding modifications of nighttime sleep schedule or limiting daytime sleep, exercise, or avoidance of bright light exposure [53].

ISWRD is the least recognized and least well characterized of the circadian rhythm sleep–wake disorders. It is not clear if ISWRD occurs as a result of, is a marker of, or contributes to the development of the neurologic, developmental, and psychiatric comorbidities with which it is associated. Given that irregular sleep patterns are often cited as a cause of institutionalization in these populations [105], a better understanding of the nature and management of this disorder could have a major impact on the health and well-being of these patients.

### Knowledge gaps specific to ISWRD

• Pathophysiology of ISWRD is poorly understood. Practical methods to explore the integrity of the light entrainment pathway (e.g. the melatonin suppression test [70] or tests of retinal function) in a clinical setting do not exist. It is not known if there are circadian abnormalities in period, phase, and/or amplitude.
• The etiology of ISWRD is poorly understood. Given the comorbidity with neurodevelopmental and neurodegenerative conditions, subtypes or perhaps phenotypes of ISWRD might exist based on different etiologies.
• There is a paucity of data regarding the true prevalence and time course of ISWRD, including: (1) what percentage of people with Alzheimer’s disease or other neurodegenerative disease also have ISWRD; (2) when along the trajectory of those diseases ISWRD develops; (3) what are the early signs of ISWRD; and (4) what is the development of ISWRD symptoms and how do they interact with progression of the underlying disease.
• There is a lack of practical diagnostic procedures including no standardized routine measure of circadian amplitude.
• There is a probable massive under-recognition of ISWRD in nursing home patients and other institutionalized populations, and no easy-to-use screening tools to aid in detection of ISWRD.
• Treatment strategies designed to address the underlying pathophysiology should be developed and tested. Currently used combination treatments need additional testing, and testing of the efficacy of individual components such as physical activity, daytime light exposure, and melatonin should be quantified. Additional treatment approaches (e.g. strategic use of caffeine or short scheduled naps to maintain wakefulness during the day, and pink noise to enhance nighttime sleep), should be considered. Functional outcomes (e.g. on cognitive function) as well as regularizing sleep and wake, should be assessed in these studies.
• The impact of ISWRD on quality of life and general health of the affected patient as well as their caregivers has not been systematically studied. The economic costs (institutionalization, caregiver burden) are likely to be high, but information is lacking.

### Recommendations and future directions specific to entrainment disorders

• Studies of how circadian clock features such as intrinsic period, the ability to entrain to photic and non-photic zeitgebers, and whether genetic factors play a role in the ability to entrain should be carried out in blind persons with and without N24SWD in order to better understand the pathophysiology of this disorder in this vulnerable population. Similar studies in sighted individuals with N24SWD should also be carried out.
• Practical methods to assess intrinsic circadian period (compared with observed period) are needed. Methods that can easily be implemented and interpreted in clinical populations are needed. To be clinically feasible, a period assessment method must be able to be obtained in the outpatient setting with minimal burden to the patient. While period assessments using clock gene expression in cultured fibroblasts obtained from a single sample have been used to assess circadian period, the period estimates are much longer than period assessed using other methods [106], and the method is not feasible for a standard clinical laboratory to implement. Serial melatonin sampling is easier to assay, but requires multiple multi-hour assessments at weekly or longer intervals, making it impractical for the standard clinical patient. The ideal circadian period assessment would combine the best aspects of both strategies. In terms of accuracy, current data suggest that the difference in period between individuals with DSWPD and controls is ~15 min, and N24SWD and controls is ~30 min so we would suggest targeting a minimum accuracy of ≤15 min [107].
• New treatment strategies for N24SWD in sighted individuals, such CBT-I, should be tested.
• Studies of how non-photic zeitgebers (e.g. exercise, food intake, and social interactions) can be used to entrain blind or sighted patients with N24SWD should be carried out.
• Treatment strategies for N24SWD in sighted individuals that test dual-purpose treatments to acutely entrain the patient and then maintain that entrainment over time are needed.
• How external desynchrony (between the central clock and the environment) and internal desynchrony (between the central clock and peripheral clocks) impact patients with N24SWD should be studied.
• Longitudinal studies of individuals with N24SWD should be carried out to better understand how clock alignment and misalignment impact mental and physical health (e.g. depression, substance use, diet, inflammation, cardiometabolic health, and pain sensitivity). These studies may provide insights into mental and physical health of individuals without N24SWD when their sleep/wake patterns are not aligned.
• Documentation of the integrity and/or abnormality of light input from the eye (e.g. cornea, retina, and ipRGC) though neural pathways to relevant brain areas as possible sources of pathophysiology in patients with N24SWD or ISWRD should be pursued.
• Standardized diagnostic procedures to assess circadian amplitude should be developed. Because this may be of greatest relevance to ISWRD where patients may have underlying neurodegenerative disorders, assessment procedures must not only consider the ability of the patient but also the caregiver to accurately complete the assessment. As such, assessment tools that allow for passive collection of data or limited number of samples would be most practical for this population.
• Methods to educate key stakeholders about ISWRD, including nursing home clinicians (e.g., nurses, social workers, physical therapists, aides), geriatricians, and neurologists, along with pediatricians and developmental specialists should be developed to improve recognition. Easy-to-use screening tools (similar to the STOP-BANG instrument for Obstructive Sleep Apnea) that can be used in these challenging populations to aid in detection of ISWRD should also be developed.
• Treatment strategies designed to address the underlying pathophysiology of ISWRD should be developed and tested. Currently used combination treatments need additional testing, as well as testing of the efficacy of individual components including physical activity, daytime light exposure, and melatonin. Additional treatment approaches (e.g. strategic use of caffeine or short scheduled naps to maintain wakefulness during the day, and methods to enhance nighttime sleep), should be tested.
• Functional outcomes (e.g. mood and cognitive function) as well as the regularization of sleep and wake times, should be assessed in studies of ISWRD.
• The impact of ISWRD on quality of life, mood, and general health of the affected patient as well as their caregivers should be studied. The economic costs (e.g. institutionalization and caregiver burden) of ISWRD and the benefits of successful treatment should be studied.

## Other Disorders: circadian rhythm disorder not otherwise specified (CRD-NOS)

Section participants: Jonathan S. Emens (leader), Phillip Cheng, Philip Gehrman, Melissa Knauert, Kiran Maski, and Phyllis C. Zee.

### Current state of the science

Individuals with CRD-NOS must meet all the general diagnostic criteria for a CRSWD without meeting the criteria for any other specific CRSWD; namely, there must exist a disturbance of sleep and/or wakefulness that is “primarily due” to endogenous circadian dysfunction or circadian misalignment that results in symptoms of insomnia and/or hypersomnolence. The intent of the diagnostic category was to capture patients with circadian based sleep–wake changes due to some other “underlying medical, neurologic, (or) psychiatric disorder.” The assumption is that, most commonly, the underlying disorder precipitates the circadian dysfunction or misalignment. Patients may show advanced, delayed, irregular or non-24-hour sleep/wake patterns. Examples include Major Depressive Disorder (with or without seasonal components), Bipolar Affective Disorder, Alzheimer’s disease, and Parkinson’s Disease.

The definition of CRD-NOS is problematic in the absence of any commercial tests or established clinical definitions of circadian dysfunction or circadian misalignment (e.g. no agreed upon normative ranges for the relationship between circadian phase and sleep timing). In other words, there is no way to determine whether symptoms of insomnia or hypersomnolence are “primarily due” to circadian pathology or whether the circadian disruption is part of the pathology of the disorder. Although such a quandary exists in the diagnosis of all CRSWD, it is especially problematic in the case of CRD-NOS due to its very broad definition.

Given the diagnostic uncertainties surrounding CRD-NOS, it is not surprising that there is little to no research on the disorder. In this setting, the very existence of this disorder is open to question. The case for this diagnostic category would be strengthened if it were demonstrated that the circadian system plays a role in the disturbances of sleep and wakefulness seen in a variety of psychiatric and medical conditions.

Multiple lines of evidence have suggested that circadian dysfunction may indeed have an impact on the development, presentation, and treatment of a range of disorders including cancer, cardio-metabolic, neurodegenerative, and psychiatric disease. These include diurnal rhythms in disease outcomes (e.g. myocardial infarction and suicide [108–110]), the adverse impact of circadian misalignment on disease outcomes or parameters important in disease development (e.g. glucose regulation and mood [111–114]), associated pathology within the circadian system itself (e.g. SCN pathology in Alzheimer’s disease [115–117]) and, more recently, changes in circadian parameters in some disease states (e.g. decreased circadian amplitude in Parkinson’s Disease [118]).

The evidence for circadian dysfunction in a wide variety of disorders implies that a diagnosis of CRD-NOS might be clinically useful, despite the uncertainties regarding diagnosis, if it resulted in circadian interventions that improved treatment outcomes among these various disorders. This remains to be tested.

### Knowledge gaps specific to CRD-NOS

• The knowledge gaps for CRD-NOS are extensive. Specifically, little to nothing is known about the epidemiology, etiology, natural history, diagnostic accuracy/validity, and treatment of CRD-NOS even when the underlying disease has been well characterized.
• The existence of CRD-NOS is in question, due to the fact that in very few disorders has any circadian dysfunction or circadian misalignment been demonstrated. With limited exceptions, alterations in circadian phase, period, phase angle of entrainment, or amplitude have not been “consistently” shown to exist in CRD-NOS. It is not known whether the underlying disorder causes the CRD-NOS; it is not known to what extent any circadian disruption is a part of the underlying “pathophysiology” of the parent disorder or is a “byproduct” of the disorder or its treatment.
• Diagnostic guidelines for CRD-NOS must be developed and must include physiological measures that demonstrate a circadian dysfunction or circadian misalignment.
• There are currently no treatment guidelines for CRD-NOS [53]. Treatments designed to correct a circadian dysfunction should also be assessed to determine whether this impacts the underlying disease’s outcomes.
• Standardized biomarkers necessary to make assessments of circadian dysfunction [119] are lacking. These biomarkers are needed to assess both misalignment between central circadian timing and social or behavioral (e.g., physical activity, eating) rhythms and between the central pacemaker and peripheral clocks.
• The study of circadian dysfunction in other disorders could move beyond CRD-NOS as currently defined to determine the extent to which circadian dysfunction or circadian misalignment contributes to a broad range of diseases. This knowledge could further circadian-based interventions that have already been shown to improve outcomes in non-CRSD populations [120, 121].

### Recommendations and future directions specific to CRD-NOS

• A large epidemiological study of CRD-NOS would be an initial step toward better characterizing the disorder.
• A study regarding the accuracy and reliability of CRD-NOS diagnoses is indicated.
• Development of an approved clinical test of circadian phase that is reimbursable by third-party payers would allow for both characterizing the disorder and selecting appropriate treatments.

## Conclusion

Given the ubiquity of circadian clocks throughout the brain and periphery, circadian disturbance is expected when there is other disease and, in turn, circadian disturbance may have far-reaching implications for every aspect of health. Although sleep and wake are the most obvious manifestation of circadian rhythm influences, the sleep and wake disturbances of CRSWD may be only one of the circadian abnormalities. An example of this is the range of adverse health outcomes associated with DSWPD and other CRSWD, including major depression, substance use, cellular aging, job loss, and school truancy. Therefore, the study of individuals with CRSWD, including assessment of internal and/or external circadian misalignment and assessment of the amplitude of central and peripheral circadian rhythms may provide unique opportunities for scientific discovery. For example, studies of this population may lead to new information on mechanisms of entrainment, novel methods for studying large cohorts of diseases in which circadian misalignment may play a role (e.g. cancer or cardiovascular disease), and innovative treatment strategies for minimizing circadian misalignment-related disorders (e.g. shiftwork).

The research and knowledge gaps described above will require cooperation and coordination from multiple parties to address. Cooperation between professional research and clinical organizations and between individual researchers and clinicians, partnerships between research groups, involvement of patient advocacy and education groups, and funding targeted towards both mechanistic understanding of CRSWD and translation of those findings through clinical, educational, and public health measures will be necessary to move forward. The Workshop participants are eager to work towards addressing the gaps and opportunities identified to improve the lives of patients with CRSWD and using the knowledge gained toward addressing circadian disruptions that co-occur with other medical and psychiatric ailments.

## Limitations

While a review of the literature was carried out in writing this White Paper, the opinions formulated here are based on experts’ opinion rather than a systematic review of the levels of evidence for the clinical management of CRSWD.

## Data Availability

No new data were generated or analyzed in support of this research.
